# Supplementary material for: Simulating rigid head motion artifacts on brain magnitude MRI data–Outcome on image quality and segmentation of the cerebral cortex
Source: PLoS One. 2024 Apr 16;19(4):e0301132. doi: 10.1371/journal.pone.0301132 (PMC11020361; doi:10.1371/journal.pone.0301132)
Supplement: S1 Fig — There is a monotonic increase for Real/Modified but not for Original. However, the change in EFC between nodding frequencies is relatively small. (DOCX) [file pone.0301132.s001.docx]

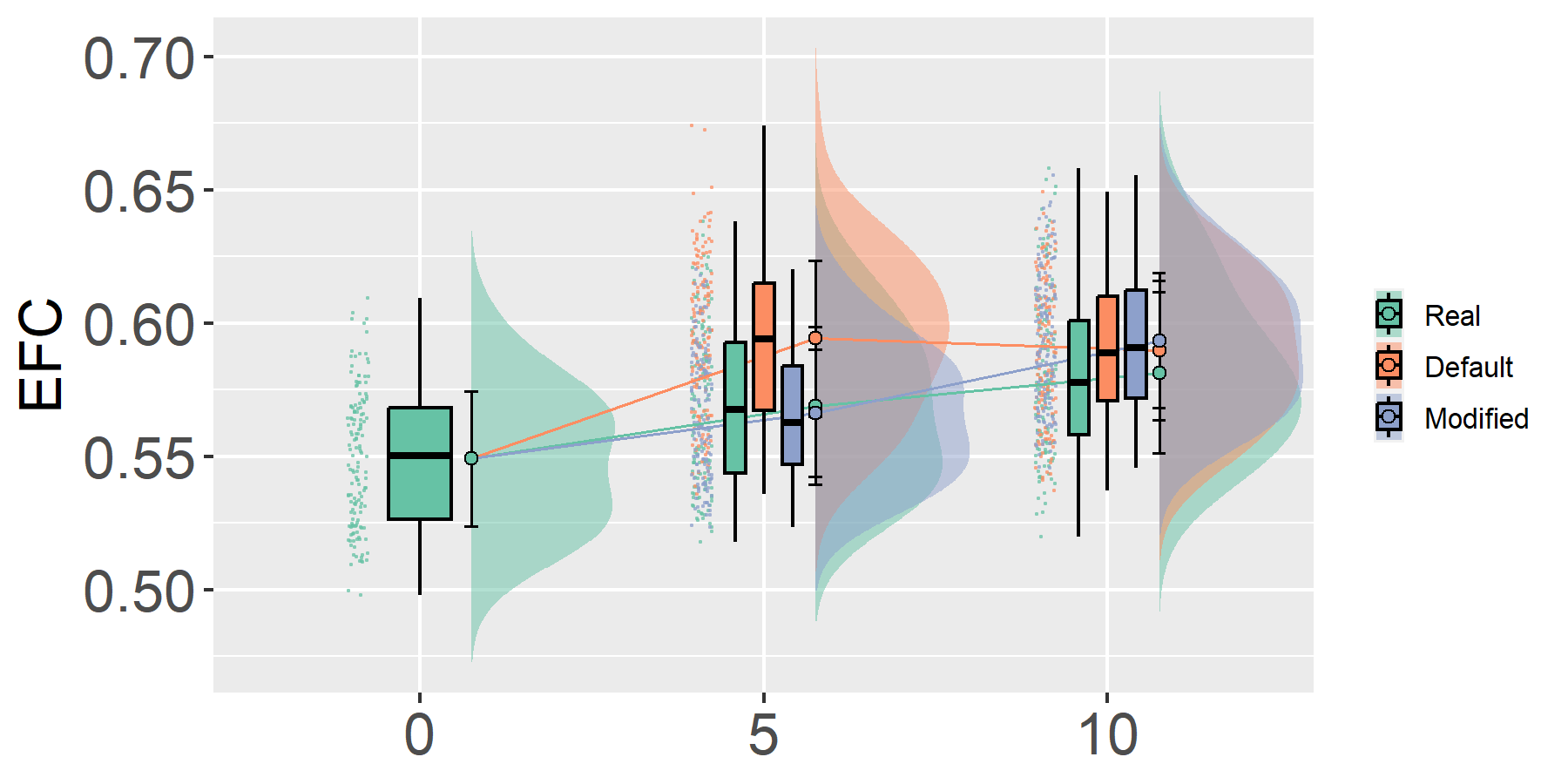


**S1 Fig.** The Entropy Focus Criterion (EFC) showed good agreement between Real/Modified. There is a monotonic increase for Real/Modified but not for Original. However, the change in EFC between nodding frequencies is relatively small.
