# Supplementary material for: Simulating rigid head motion artifacts on brain magnitude MRI data–Outcome on image quality and segmentation of the cerebral cortex
Source: PLoS One. 2024 Apr 16;19(4):e0301132. doi: 10.1371/journal.pone.0301132 (PMC11020361; doi:10.1371/journal.pone.0301132)
Supplement: S2 Fig — (DOCX) [file pone.0301132.s002.docx]

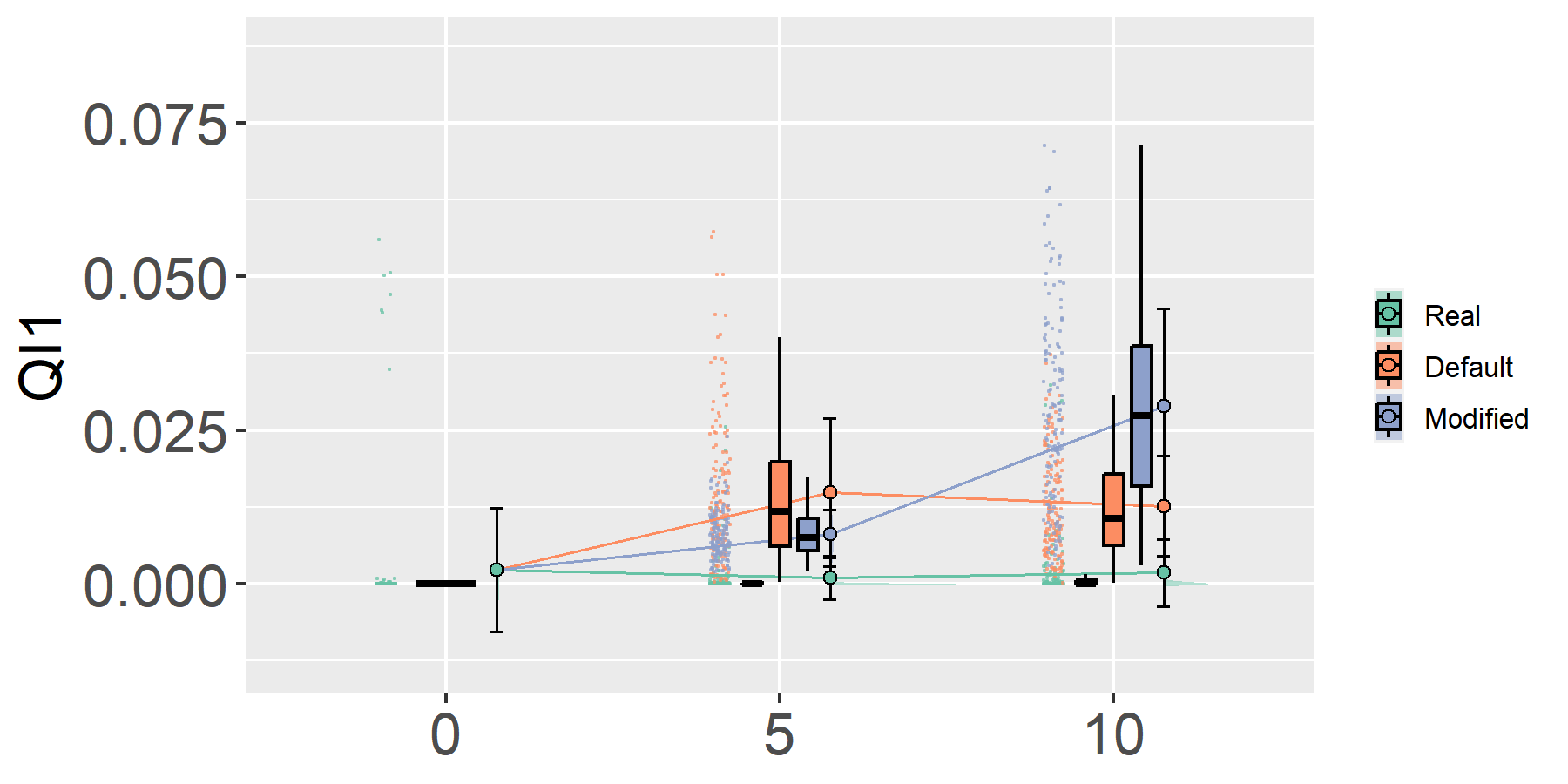


**S2 Fig**. The quality index, QI1, showed inconclusive results, likely because of its dependency on background pixels.
