# Supplementary material for: Simulating rigid head motion artifacts on brain magnitude MRI data–Outcome on image quality and segmentation of the cerebral cortex
Source: PLoS One. 2024 Apr 16;19(4):e0301132. doi: 10.1371/journal.pone.0301132 (PMC11020361; doi:10.1371/journal.pone.0301132)
Supplement: Attachment — Submitted filename: Response to Reviewers.docx [file pone.0301132.s004.docx]

# Response to Reviewers

Reviewer comments are in blue, replies in black and quotations from the manuscript are denoted by dashed lines.

**Reviewer 1 (R1)**

**R1.1 -** More detailed information is needed regarding the processing of the DICOMs done via TorchIO.

We have added more detailed information regarding the loading of data and the data structure within TorchIO. We’ve also included a reference to the TorchIO documentation for the convenience of the reader, in case a more extensive explanation is needed. Please note that no additional processing was performed on the NIfTI data other than the motion simulations described in the manuscript. Perhaps this point also related to R1.2 concerning the “RandomMotion” function. Please also see our response there. Methods->Motion simulations-> 1^st^ paragraph now reads as:

--------------------------------------------------------------------------------------------------------------------------------------

The MR-ART NIfTI files were loaded into TorchIO as a ‘SubjectsDataset’ consisting of 148 ‘Subject’ objects which each contained a single image volume represented by an image class. From here, additional preprocessing and augmentation can be performed. Detailed information about the general TorchIO data processing can be found here: https://torchio.readthedocs.io/index.html.

--------------------------------------------------------------------------------------------------------------------------------------

**R1.2** - The “RandomMotion” function generates motion randomly, as stated in the work. How was this addressed? Was the generation repeated for the same range of values to have more variability?

The RandomMotion function was not repeated to have more variability for the same subject. The function was applied twice on the motion-free image of each subject. The 1^st^ time, 5 discrete motions were simulated (Ori5). The 2^nd^ time, 10 discrete motions were simulated (Ori10). Each time, a unique discrete motion was simulated: a pitch, a yaw, and a roll. Each of these three variables were randomly selected from the defined range of 0-15°. The below table shows an example of what this could look like for a particular subject.

Ori5

| Discrete movement | #1 | #2 | #3 | #4 | #5 |
| --- | --- | --- | --- | --- | --- |
| Pitch [°] | 0 | 13 | 7 | 5 | 8 |
| Yaw [°] | 14 | 1 | 4 | 5 | 12 |
| Roll [°] | 7 | 14 | 15 | 10 | 14 |

Ori10

| Discrete movement | #1 | #2 | #3 | #4 | #5 | #6 | #7 | #8 | #9 | #10 |
| --- | --- | --- | --- | --- | --- | --- | --- | --- | --- | --- |
| Pitch | 7 | 0 | 10 | 3 | 13 | 15 | 3 | 7 | 4 | 12 |
| Yaw | 3 | 9 | 1 | 15 | 14 | 12 | 5 | 1 | 8 | 3 |
| Roll | 15 | 10 | 7 | 6 | 1 | 2 | 13 | 14 | 9 | 14 |

The discrete motions for both motion severity levels (5 and 10 discrete movements) were thus never the same for any subject but rather randomly different. This is now clearly stated in the manuscript together with a condensed version of the above example (Methods->Motion simulations->1^st^ paragraph):

--------------------------------------------------------------------------------------------------------------------------------------

For an example subject, 5 simulated movements with a rotational range of 0-15° could result in a pitch =4°/13°/15°/8°/5°, a yaw = 13°/0°/6°/0°/5°, and a roll = 0°/2°/7°/4°/11°. New numbers are generated each time the ‘RandomMotion’ function is applied, leading to a wide range of generated motion artifacts in between subjects in the generated dataset.

--------------------------------------------------------------------------------------------------------------------------------------

**R1.3** - The Motion corruption using RandomMotion looks worse than the original image. A comparison of the motion parameters should be performed and discussed.

It is correct that Ori5/Ori10 displays an overall worse image quality compared to Real5/Real10 in Figures 3 and 4 as mentioned in Results->Qualitative characteristics of real and simulated motion artifacts. Unfortunately, a direct comparison of motion parameters in the individual case is difficult since the exact motion paradigm is not known in the MR-ART dataset (Methods->The MR-ART dataset and Discussion->2^nd^ paragraph). It is also regrettably the case that motion simulation parameters on the individual level were not saved for Ori5/Ori10. This is now clearly stated (Results->Qualitative characteristics of real and simulated motion artifacts):

--------------------------------------------------------------------------------------------------------------------------------------

Note that the exact motion paradigm is not known on an individual level for Real5/Real10 and Ori5/Ori10.

--------------------------------------------------------------------------------------------------------------------------------------

We have also added a section in the Discussion reflecting on the qualitative differences that can be seen in the example subject (Discussion->2^nd^ paragraph):

--------------------------------------------------------------------------------------------------------------------------------------

The improved agreement was also visible on a qualitative level, where the characteristic ringing artifacts of the MR-ART dataset could be much better simulated using the modified simulations. Here, the random behavior of the original simulations instead results in blurring and perceived overall degraded image quality.

--------------------------------------------------------------------------------------------------------------------------------------

**R1.4** - How do your findings regarding the segmentation tools relate with the current literature? One source could be <https://discovery.ucl.ac.uk/id/eprint/10174895/>.

We thank the reviewer for this suggestion. We have included the reference to the study by Kemenczky P et al., Scientific reports 2022, in addition to our previous discussion (line 360) and now discuss the results by van Nederpelt et al., Neuroradiology 2023 in more detail (Discussion->4^th^ paragraph):

--------------------------------------------------------------------------------------------------------------------------------------

This hypothesis is supported by previous work which reported higher total GM volumes using SynthSeg (albeit not SynthSeg+) compared to FreeSurfer, SAMSEG, and FastSurfer [32].

--------------------------------------------------------------------------------------------------------------------------------------

and

--------------------------------------------------------------------------------------------------------------------------------------

van Nederpelt et al. [32] reported markedly lower intra-class correlation coefficients on data from repeated measurements when using FreeSurfer, compared to SAMSEG, FastSurfer, and SynthSeg (albeit not SynthSeg+). This could be related to the motion robustness analysis performed in our study, although here it is SynthSeg+ that stands out in terms of high DSCs.

--------------------------------------------------------------------------------------------------------------------------------------

**R1.5** - In various parts of the manuscripts, references are reported only using the name of the first author (e.g. Kemenczky et al.), without reporting the actual reference number. References need to be harmonized and written to be easily searched by the reader e.g. either adding the reference number or the year of the publication.

For better visibility and accessibility, the reference number is now included directly after the author’s name rather than at the end of the sentence.

**Reviewer 2 (R2)**

**R2.1** - Although it is a useful analysis, the results are not surprising. The results would be more meaningful if the authors could provide a justification for why the original TorchIO library was chosen for MRI motion simulation given its limitations.

The “random motion” approach is not unique to TorchIO and has been implemented elsewhere. For instance, *Shaw R, et al., IEEE transactions on medical imaging. 2020;39(9):2881-92* and *Graham MS, et al., Neuroimage. 2016;125(15):1079-94*. TorchIO is a user-friendly framework for standardization of medical image data pre-processing, which motivates using its in-built functionality for motion simulations as reference for machine learning purposes. Further, the random feature of the motion simulations in TorchIO follows the rationale for general image data augmentation, which is designed to artificially increase training data variability. It is thus warranted to investigate the impact of removing the random element in the motion simulations and to explore to what extent this makes the resulting images more comparable to real-life data. We agree that the justification for our approach was not made sufficiently clear in the original text. Thus, the following has been added to the Introduction (Introduction->3^rd^ paragraph):

--------------------------------------------------------------------------------------------------------------------------------------

TorchIO facilitates the standardization of medical image data augmentation for deep learning projects. Included in its library are standard augmentation techniques, such as flipping and spatial transformations, as well as MRI-specific modifications, such as those for bias field and motion. Augmentation functions are typically random, generating different results each time they are called [16]. The MRI-specific simulations in TorchIO follow this convention and are thus not designed to simulate specific motion paradigms and/or pulse sequences. This “random motion” approach is not unique to TorchIO and has been implemented in other studies [6, 17-18].

--------------------------------------------------------------------------------------------------------------------------------------

and (Discussion->1^st^ paragraph)

--------------------------------------------------------------------------------------------------------------------------------------

It should be noted that the random characteristic of the original motion simulations in TorchIO is not an inherent weakness of the framework but rather an effective feature to artificially increase training data variability for deep learning applications. However, when a specific motion paradigm and/or pulse sequence is to be simulated, modifications as the ones described here become necessary.

--------------------------------------------------------------------------------------------------------------------------------------

**R2.2** - There are several places in the manuscript where the authors speculate about other published work based on their findings (Methods>Image Quality 2^nd^ paragraph, Discussion 2^nd^ and 6^th^ paragraphs.) The information should be rewritten to list the facts and remove speculation.

The speculative statements have been removed.

The text in Methods->Image quality->2^nd^ paragraph now reads as follows:

--------------------------------------------------------------------------------------------------------------------------------------

Before MRIQC processing, a defaced mask was created for each subject, based on the NoMotion image, and applied to the motion simulated images. This removed pixels that were not present in the original masked dataset and thus facilitated a more correct comparison. Thereafter, all 148x7-8=1028 images were run through the MRIQC pipeline. Processing was performed using an MRIQC Docker container, version 23.1.0.

--------------------------------------------------------------------------------------------------------------------------------------

The corresponding section in the Discussion->2^nd^ paragraph was also changed to:

--------------------------------------------------------------------------------------------------------------------------------------

The IQMs QI1 and QI2 showed inconclusive results across different (even real) nodding frequencies. It should be noted that the data was defaced making the air background-based analysis not possible [22].

--------------------------------------------------------------------------------------------------------------------------------------

And the sentence the reviewer refers to in the Discussion->6^th^ paragraph now reads as follows:

--------------------------------------------------------------------------------------------------------------------------------------

Kemenczky et al. [33] used radiologist image quality rating scores, available in the published dataset, instead of IQMs to evaluate the motion robustness of deep learning-based brain segmentation tools compared to FreeSurfer.

--------------------------------------------------------------------------------------------------------------------------------------

**R2.3** - In the Conclusion, the authors state that their simulation of realistic motion artifacts helps to mitigate biases between study groups and studies. There is no data in the paper to support this claim and it should be removed. Hopefully, the authors can apply their method and show such data in the future.

We thank the reviewer for their observation and have changed the relevant sentence as follows:

--------------------------------------------------------------------------------------------------------------------------------------

Future work could use this simulation approach to mitigate biases in the distribution of motion artifacts between study groups and to provide an unbiased foundation for the training of machine learning based models.

--------------------------------------------------------------------------------------------------------------------------------------

**R2.4** - Although the proposed simulation more closely matches the real data in IQM distribution, they are still quite different, as seen in Fig. 5. This is especially true as the motion increases from 5 to 10 movements. The authors discuss limitations of their simulated data to match the real motion data. There are additional limitations, in addition to not being multi-channel k-space data or subjects having variable movement. For example, real motion can occur during different steps in the phase-encode along two axes. He MR-ART data uses an acceleration factor of GRAPPA=2. This type of real data can also have an interaction between the GRAPPA kernel used in the reconstruction and the motion corrupted k-space data. The authors could add a discussion about some of the other factors that produce artifacts in real motion corrupted MRI data, if they could be simulated in k-space based data, and if they could be simulated with magnitude MRI data.

A paragraph discussing the limitations of a magnitude data-based approach for motion simulations regarding parallel imaging (GRAPPA in particular), receive field sensitivity, translational (rather than rotational) motion was added to Discussion->5^th^ paragraph:

--------------------------------------------------------------------------------------------------------------------------------------

Multi-channel phased arrays are typically employed for parallel imaging [36]. In the MR-ART dataset, a GRAPPA factor of 2 was applied, which means that every other k-space line is not measured but synthesized using weighting factors based on the measured data and indirect sensitivity measurements [37]. The interplay between the timing of discrete movements and the calculation of these weighting factors cannot be simulated using magnitude data alone. This is especially true when considering that receive sensitives are highly variable and depend on the position of the subject. Further, we have here focused only on simulating rotational motion where a rotation in image space results in an identical rotation in k-space. Translational motion will instead result in a linear phase ramp according to the Fourier shift theorem [38]. Although our suggested approach does not directly manipulate the pseudo k-space, it would be interesting to examine how a magnitude-based approach would compare to real-world data acquired when using a translational instead of rotational motion approach.

--------------------------------------------------------------------------------------------------------------------------------------
