# Supplementary material for: Simulating rigid head motion artifacts on brain magnitude MRI data–Outcome on image quality and segmentation of the cerebral cortex
Source: PLoS One. 2024 Apr 16;19(4):e0301132. doi: 10.1371/journal.pone.0301132 (PMC11020361; doi:10.1371/journal.pone.0301132)
Supplement: Attachment — Submitted filename: Response to Reviewers.docx [file pone.0301132.s005.docx]

# Response to Reviewers

# PONE-D-23-32888_R2

2024.03.08

Reviewer comments are in blue, replies in black and quotations from the manuscript are denoted by dashed lines.

**Reviewer 1 (R1)**

All the suggested phrase changes have been incorporated exactly as suggested. We appreciate the precise wording by the reviewer which made revision very convenient. One exception is the sentence below which was re-written to clearly state that three masks are used to create a single mask of the cortical gray matter:

--------------------------------------------------------------------------------------------------------------------------------------

As BrainSuite does not directly output a cortical segmentation, we derived a NIfTI volume that could be compared to the output of the other segmentation tools by combining three masks: A mask of the boundary between white matter and cortical gray matter, a mask of the total gray matter (cortical and deep gray matter) based on a GM probability map (pixels with >50% probability of belonging to GM was kept), and a mask of the cerebrum.

--------------------------------------------------------------------------------------------------------------------------------------

**Reviewer 3 (R3)**

**R3.1** - Linguistically, I would recommend changing the first paragraph in the results section as a nuance: It would be more precise if instead of "ringing artifacts were represented" you would use "ringing artifacts were mimicked closer to the original" and instead of "instead showed a general blurring" you would rather use create or appeared as... (both starting from line 251).

The relevant sentences now reads as:

--------------------------------------------------------------------------------------------------------------------------------------

When performing the simulations (Fig 1), these ringing artifacts were mimicked closer to the real motion-corrupted images with the modified simulations (Mod5, Mod10). Images created with the original simulations (Ori5, Ori10) did not display these ringing artifacts as clearly but instead appeared as more blurred and with a worse overall image quality.

--------------------------------------------------------------------------------------------------------------------------------------
